# Interactive Effects of Irrigation Amount, Irrigation Salinity, and Nitrogen Rate on Soil Enzyme Activities and Maize (*Zea mays* L.) Yield Under Mulched Drip Irrigation

**DOI:** 10.3390/plants15142165

**Published:** 2026-07-14

**Authors:** Dongmei Han, Qijin Zhou, Zhanli Ma, Zhenhua Wang, Jinzhu Zhang, Yunkai Li

**Affiliations:** 1 College of Water Conservancy & Architectural Engineering, Shihezi University, Shihezi 832000, China; 2 Technology Innovation Center for Agricultural Water and Fertilizer Efficiency Equipment of Xinjiang Production & Construction Corps, Shihezi 832000, China; 3 Teaching and Research Office, Shihezi Engineering Technical College, Shihezi 832000, China; 4 Key Laboratory of Modern Water-Saving Irrigation of Xinjiang Production & Construction Group, Shihezi University, Shihezi 832000, China; 5 College of Water Resources and Civil Engineering, China Agricultural University, Beijing 100083, China; 6 Engineering Technology Center in the Corps for Comprehensive Utilization of Saline-Alkali Land, Shihezi University, Shihezi 832000, China

**Keywords:** *Zea mays* L., brackish water irrigation, nitrogen application, soil salinization, soil enzymes, maize yield, resource use efficiency, arid region, TOPSIS

## Abstract

Brackish water and fertilizer must be used more efficiently to sustain maize production in arid regions affected by freshwater scarcity and soil salinization. Yet, the coupled effects of irrigation amount, irrigation salinity, and nitrogen application on soil biochemical processes and maize productivity under mulched drip irrigation remain unclear. We conducted a two-year field experiment (2023–2024) in a sandy loam field in arid northwestern China to evaluate irrigation amount (450 mm, W1; 675 mm, W2), irrigation salinity (1.3 dS m^−1^, S1; 3.5 dS m^−1^, S2; 5.7 dS m^−1^, S3), and nitrogen rate (225 kg ha^−1^, N1; 330 kg ha^−1^, N2; 435 kg ha^−1^, N3). The experiment measured four soil enzyme activities, soil physicochemical properties, and maize yield. Higher irrigation increased soil water storage by about 27%, but it also increased salt accumulation by about 9% across the two seasons. Increasing irrigation salinity from S1 to S3 reduced urease, alkaline phosphatase, protease, and sucrase activities by 8.52%, 6.72%, 13.75%, and 3.65%, respectively, suggesting weaker nutrient turnover and uptake under salinity stress. By contrast, the higher irrigation amount (W2) increased these activities by 10.27%, 9.73%, 11.08%, and 9.47%, respectively. Structural equation modeling indicated that soil salt accumulation constrained yield mainly by reducing grain number rather than 1000-grain weight. Although higher irrigation increased mean yield, the integrated evaluation identified W1S2N2 as the best combination. This regime produced the highest grain yield (18,355.76 kg ha^−1^) and irrigation water productivity (40.79 kg mm^−1^), which were 82.17% and 155.26% higher than the corresponding lowest values, while maintaining relatively high partial factor productivity of nitrogen (55.62 kg kg^−1^) in 2024. These findings suggest that controlled deficit irrigation (450 mm) and moderate nitrogen input (330 kg ha^−1^) are recommended for long-term sustainability in arid regions, but longer-term salinity monitoring is required before a broad recommendation can be made when using brackish water irrigation.

## 1. Introduction

The shortage of freshwater resources deeply limits agricultural productivity and sustainability across arid and semi-arid regions. This constraint is particularly acute in Xinjiang, northwestern China, where crop production depends heavily on irrigation and the agriculture sector consumes over 90% of regional freshwater resources. Maize (*Zea mays* L.) is important for grain production in this region, but its sensitivity to water deficits and salt stress increases production risk when freshwater supplies are limited [1,2]. At the same time, soil salinization is expanding, as saline–alkali land accounts for more than one-third of the cultivated area in Xinjiang [3,4,5]. Maintaining maize yield while protecting soil resources, therefore, requires irrigation strategies that can manage both water shortages and salinity.

Xinjiang also has substantial brackish and saline water reserves. Shallow groundwater, for example, can account for nearly 70% of total water resources in parts of the region, but it remains underused in agriculture [5]. Brackish water (2–5 g L^−1^) is therefore considered a potential substitute for freshwater irrigation in water-scarce areas [6], provided that salt input and leaching are carefully managed. Experience from other dryland systems shows that saline or brackish irrigation can sustain crop production when salts are controlled. In Israel, saline irrigation has been used on light- and medium-textured soils together with sufficient leaching [7]. In the southwestern United States, cotton yields under moderately saline irrigation matched or exceeded those under freshwater when appropriate management was implemented [8]. However, the use of brackish water in Xinjiang is still limited by the lack of irrigation regimes that simultaneously balance water supply, salt accumulation risk, and crop productivity.

Nitrogen (N) is a key factor supporting crop growth, while the input of N also brings more complexity for the use of brackish water. N supply generally regulates maize leaf area, root growth, and photosynthetic capacity [9]. Therefore, the crop response to N strongly depends on soil water availability and salt distribution in the root zone [10,11]. Field studies on drip-irrigated maize in arid northwestern China show that moderate N application with an optimized irrigation amount can improve grain yield and water productivity, whereas excessive N gives little additional yield and reduces nitrogen use efficiency (NUE) [12]. Similar patterns in drip-irrigated cotton in Xinjiang indicate that poorly matched N and irrigation inputs can reduce resource efficiency rather than increase productivity [13].

Soil enzymes are active in the plant rhizosphere environment, and they can convert unavailable organic compounds into nutrient forms, thus improving soil fertility. Urease (URE), alkaline phosphatase (ALP), protease (PRO), and sucrase (SUC) regulate key steps in N mineralization, phosphorus turnover, and organic matter decomposition. Specifically, URE and PRO are closely related to nitrogen transformation, with URE catalyzing urea hydrolysis and PRO participating in the decomposition of proteinaceous organic nitrogen. ALP reflects the mineralization potential of organic phosphorus, while SUC is associated with labile carbon turnover and microbial energy supply [14,15,16]. Their activities are important biochemical indicators that respond to changes in the soil environment. These enzymes may respond differently to salinity stress because of their distinct catalytic functions, substrate sources, and microbial origins. Studies have shown that high salinity can inhibit enzyme activity by reducing microbial biomass and limiting substrate diffusion [17]. However, the magnitude of inhibition may vary among enzymes. For example, C- and N-cycling enzymes such as SUC, PRO, and URE are often closely linked to microbial metabolism and substrate availability, whereas ALP is more strongly regulated by soil phosphorus demand and nutrient limitation [18,19,20]. Suitable nitrogen supply may partly alleviate salt-induced constraints by stimulating microbial growth, improving nutrient availability, and promoting enzyme production [21,22,23,24]. Soil moisture status also affects enzyme kinetics and substrate availability. Nevertheless, there is little evidence focusing on how enzyme activities respond to the combined effects of irrigation amount, irrigation salinity, and N input under MDI, especially when brackish water is used. Clarifying these responses can connect soil water–salt–nutrient dynamics with crop yield formation and resource use efficiency.

Mulched drip irrigation (MDI) is widely used in arid and semi-arid regions because it reduces soil evaporation, redistributes salts away from the root zone, and improves crop water and nitrogen use efficiencies [25,26,27]. Although numerous studies have examined pairwise interactions such as water–nitrogen coupling [18,19,20,21,28,29] or salinity–nitrogen interactions under drip irrigation systems, these double-interaction approaches may not fully capture the non-additive effects generated by the simultaneous variation in irrigation amount, irrigation water salinity, and nitrogen application rate. Recent evidence has shown that optimizing these three factors together can improve nitrogen uptake and final fiber yield in cotton under MDI [19,30]. However, such triple-factor studies remain limited, particularly for maize production systems. Moreover, previous work has mainly emphasized crop yield, nitrogen uptake, or soil water–salt–nitrogen transport, whereas the responses of soil enzyme activities and their mechanistic links with maize productivity under W × S × N interactions remain insufficiently understood.

The objectives of the present study are to (1) quantify the interactive effects of these three factors on soil physicochemical and biochemical properties and (2) determine how they jointly regulate maize yield and resource use efficiency in an arid environment. We hypothesized that (1) moderate irrigation salinity and N input would maintain higher enzyme activity and maize productivity, (2) irrigation amount would mitigate salinity stress but could also increase salt accumulation when saline water was applied, and (3) yield loss under salt accumulation would be expressed mainly through reduced grain number rather than reduced grain weight. The results are intended to support integrated water–fertilizer–salinity management for sustainable maize production in saline agricultural regions.

## 2. Results

### 2.1. Soil Water Storage

W and S had significant main effects on SWS during the 2023 and 2024 maize growing seasons (*p* < 0.05). By contrast, the two-way interactions (W × N, W × S, and N × S), and the three-way interaction (W × N × S), did not significantly affect SWS (Table 1). Because more water was applied, SWS under W2 was consistently higher than under W1 in both years. Across the 0–100 cm soil profile, W2 increased SWS by 27.30% in 2023 and 27.07% in 2024 relative to W1. SWS also increased with irrigation salinity at the 0–40, 40–100, and 0–100 cm. The highest SWS occurred under S3, exceeding that under S2 and S1 by 6.58% and 13.28%, respectively. N application did not significantly affect SWS in 2023, but it did affect SWS in 2024 (*p* < 0.05), when the lowest SWS was recorded under N2.

### 2.2. Soil Salt Accumulation

In both years, the three factors had significant main effects on SSA (*p* < 0.05) (Table 2). The W × S interaction was also significant (*p* < 0.05), whereas W × N, N × S, and W × N × S were not (*p* > 0.05). Overall, SSA increased as irrigation amount, N application rate, and irrigation salinity increased. Compared with W1, W2 increased SSA in the 0–100 cm soil profile by 9.16% in 2023 and 8.90% in 2024. Compared with N1, N2 and N3 increased SSA by 8.94% and 19.36% in 2023, and by 16.11% and 25.00% in 2024, respectively. The large F values for irrigation salinity also show that salinity was the dominant source of variation in SSA, exceeding the effects of irrigation amount and N rate. Compared with S1, S2 and S3 increased SSA by 50.78% and 109.90% in 2023, and by 47.86% and 103.63% in 2024, respectively, across the 0–100 cm soil profile.

### 2.3. Soil Total Nitrogen

**Figure 1:**
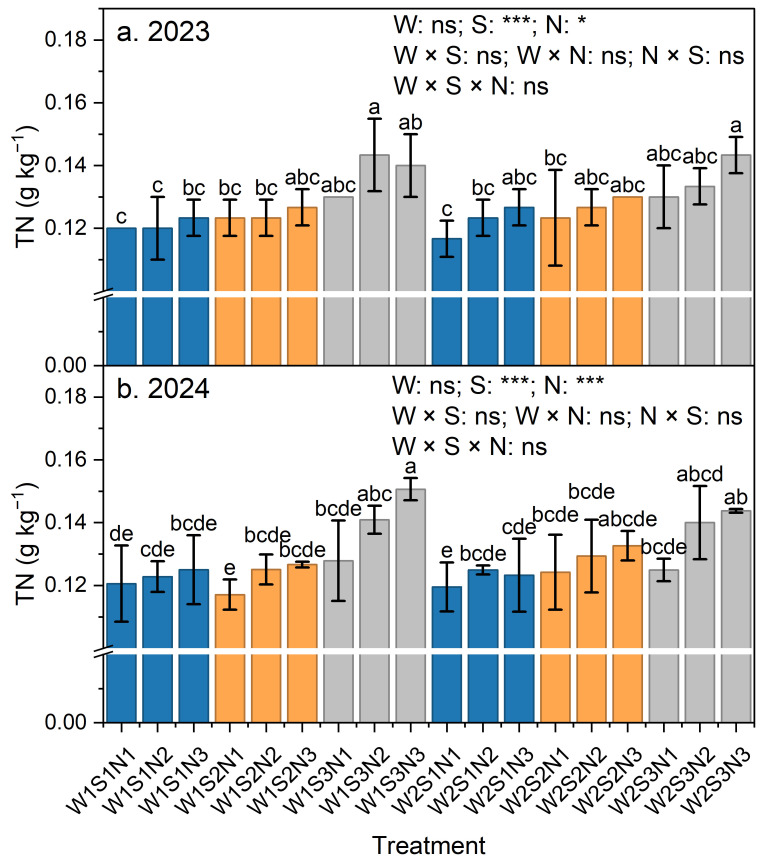
Effect of different irrigation amounts (W), irrigation salinities (S), and nitrogen application rates (N) on soil total nitrogen (TN) content after maize was harvested in 2023 (**a**) and 2024 (**b**). Values are presented as mean ± standard deviation. Different lowercase letters in the figure indicate significant differences between treatments at the 0.05 level. *, *** indicate significant levels at *p* < 0.05 and *p* < 0.001, respectively; “ns” indicates no significant difference.

Soil TN showed a consistent increase with N rate across the two-year experiment (Figure 1). TN ranged from 0.12 to 0.14 g kg^−1^ in 2023 and from 0.12 to 0.15 g kg^−1^ in 2024. The highest TN occurred under W2S3N3 in 2023 and W1S3N3 in 2024. ANOVA showed that S and N significantly affected soil TN (*p* < 0.05). W and all two-way and three-way interactions among the three factors had no significant effect.

### 2.4. Soil Enzyme Activities

**Figure 2:**
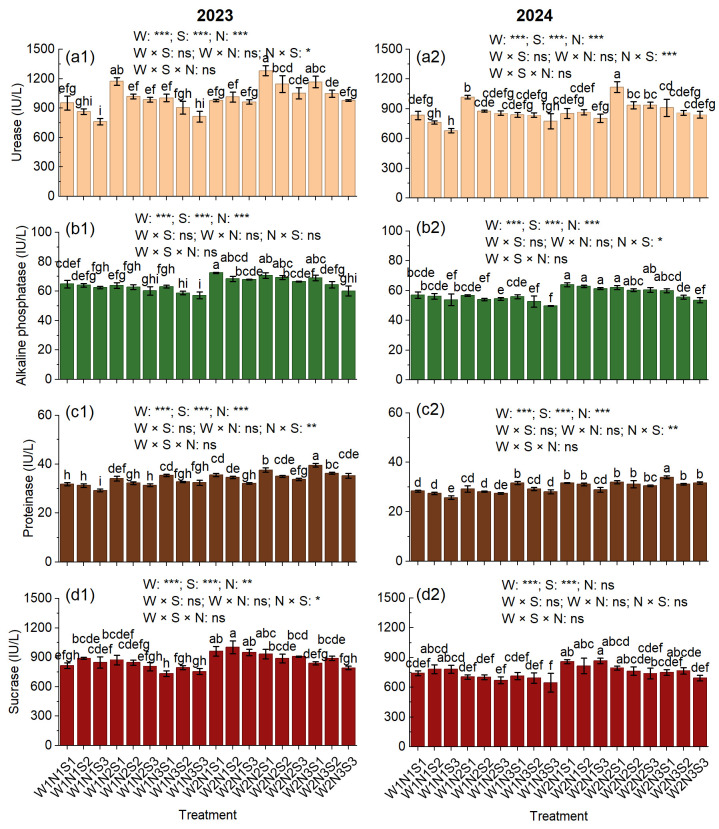
Effect of different irrigation amounts (W), irrigation salinities (S), and nitrogen application rates (N) on the activities of urease (URE), alkaline phosphatase (ALP), proteinase (PRO), and sucrase (SUC) after maize was harvested in 2023 (**a1**–**d1**) and 2024 (**a2**–**d2**). Values are presented as mean ± standard deviation. Different lowercase letters in the figure indicate significant differences between treatments at the 0.05 level. *, **, *** indicate significant levels at *p* < 0.05, *p* < 0.01 and *p* < 0.001, respectively; “ns” indicates no significant difference.

After maize harvest, W, S, and N all significantly affected URE, ALP, PRO, and SUC activities (*p* < 0.001, Figure 2). All four enzyme activities increased under the higher irrigation amount, indicating that better soil moisture improved the biological environment for microbial activity in this arid field. Relative to W1, W2 increased URE, ALP, PRO, and SUC activities by 10.27%, 9.73%, 11.08%, and 9.47%, respectively. In contrast, higher irrigation salinity suppressed enzyme activities. From S1 to S3, URE, ALP, PRO, and SUC activities decreased by 8.52%, 6.72%, 13.75%, and 3.65%, respectively.

The enzymes responded differently to the N gradient. PRO activity increased as N application increased, similar to its response to irrigation amount. ALP and SUC activities decreased with greater N addition. URE showed a nonlinear pattern, increasing at first and then decreasing, with the highest activity recorded in W2N2S1, with values of 1281.37 IU L^−1^ in 2023 and 1118.47 IU L^−1^ in 2024, respectively. Among the interaction terms, only N × S significantly affected enzyme activities, suggesting that salinity altered how N regulated soil enzymatic processes.

### 2.5. Maize Productivity

W, S, N, and their interactions significantly affected maize grain yield, IWP, and PFPN in both 2023 and 2024 (Tables S1 and S2). For the single-factor effects, W2, N2, and S2 generally produced the highest grain number, grain yield, and IWP in both seasons, whereas PFPN was highest under N1. Among the three-factor combinations, W1S2N2 produced the highest maize grain yield and IWP in both years (Table 3), reaching 17,663.86 kg ha^−1^ and 39.25 kg mm^−1^ in 2023, and 18,355.76 kg ha^−1^ and 40.79 kg mm^−1^ in 2024, respectively. The maximum PFPN was observed under W1S2N1, with values of 68.62 kg kg^−1^ in 2023 and 69.44 kg kg^−1^ in 2024. In contrast, W1S3N3 produced the lowest maize grain yield, with 10,272.18 kg ha^−1^ in 2023 and 10,076.28 kg ha^−1^ in 2024, as well as the lowest PFPN, with 23.61 kg kg^−1^ in 2023 and 23.16 kg kg^−1^ in 2024. The lowest IWP occurred under W2S3N1, reaching only 17.42 kg mm^−1^ in 2023 and 15.98 kg mm^−1^ in 2024. Compared with these lowest performing treatments, the treatments with the highest values increased grain yield, IWP, and PFPN by 71.96%, 125.32%, and 190.64% in 2023, respectively, and by 82.17%, 155.26%, and 199.83% in 2024, respectively.

### 2.6. Grain Yield Formation Pathways Analysis and Treatment Performance

**Figure 3:**
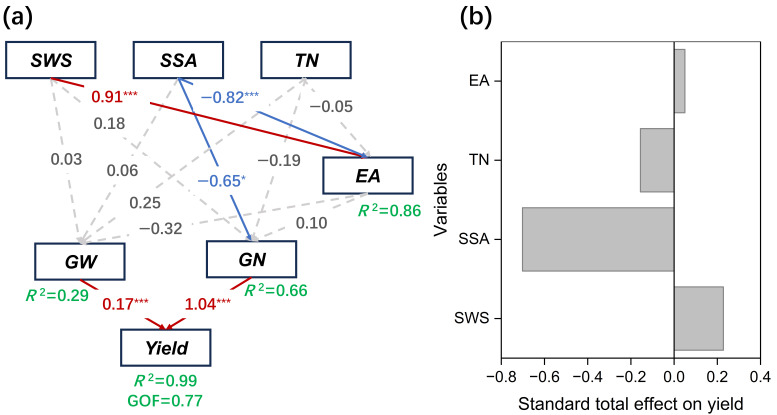
Direct effects (**a**) and total effects (**b**) of soil water storage (SWS), soil salt accumulation (SSA), total nitrogen (TN), enzyme activity (EA), 1000-grain weight (GW), and grain number (GN) on maize grain yield. Values alongside the path arrows are the path coefficients (direct effects). Red and blue arrows denote positive and negative effects, respectively; solid lines and dashed lines indicate significant and non-significant paths, respectively. Significance levels: *, *p* < 0.05; ***, *p* < 0.001.

Structural equation modeling (SEM) was used to identify the pathways through which the combined irrigation amount, irrigation salinity, and N rate affected maize yield (Figure 3). The model showed a strong fit (GOF = 0.77) and explained 99% of yield variation (Figure 3a). Both 1000-grain weight (GW) and grain number (GN) had significant positive effects on yield (*p* < 0.001). GN had a much larger direct effect (path coefficient = 1.04) than GW (0.17). Among soil factors, SSA had the strongest negative direct effect on GN (−0.65, *p* < 0.05), indicating that salt accumulation reduced yield mainly by suppressing GN rather than GW. SSA also had the largest total negative effect on yield (−0.702), followed by SWS (0.228), TN (−0.156), and EA (0.050) (Figure 3b). For EA regulation, SWS had a significant positive direct effect (0.91, *p* < 0.001), whereas SSA had a significant negative direct effect (−0.82, *p* < 0.001).

**Figure 4:**
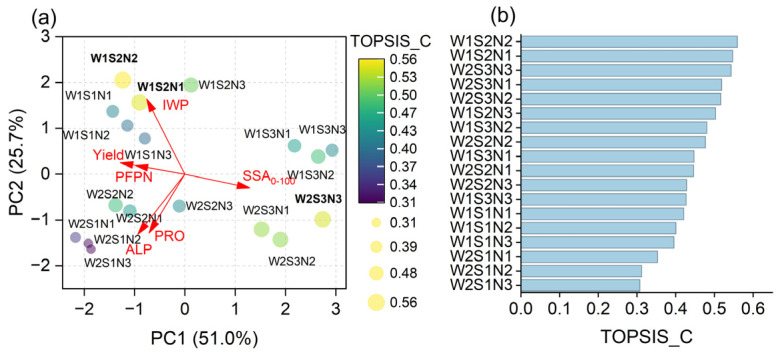
Principal component analysis (PCA)-entropy-TOPSIS results. The left panel (**a**) shows the PCA biplot based on six indicators. Point colors and size represent TOPSIS scores (C_i_), and the top three treatments are highlighted in bold. The right panel (**b**) shows the TOPSIS ranking. SSA_0–100_: soil salt accumulation at the depth of 0–100 cm; ALP: alkaline phosphatase activity; PRO: protein activity; Yield: maize grain yield; IWP: irrigation water productivity; PFPN: nitrogen partial factor productivity.

The PCA identified SSA_0–100_ (0.442), ALP (−0.442), and PRO (−0.573) as the soil indicators with the highest absolute loadings in principal components with eigenvalues greater than 1 (Table S3). These indicators, together with maize grain yield, IWP, and PFPN, were used for comprehensive evaluation. Entropy weights followed the order SSA_0–100_ (0.235) > IWP (0.202) > yield (0.178) > PFPN (0.149) > ALP (0.125) > PRO (0.111) (Table S4). In the six-indicator PCA, PC1 and PC2 explained 51.0% and 25.7% of the total variance, respectively (Figure 4a). The entropy-TOPSIS closeness coefficients separated the treatments clearly, with W1S2N2 having the highest score (C_i_ = 0.559), followed by W1S2N1 (C_i_ = 0.547) and W2S3N3 (C_i_ = 0.543, Figure 4b).

## 3. Discussion

### 3.1. Effects of Irrigation Amount, Irrigation Salinity, and Nitrogen Rate on Soil Water-Salt Dynamics

The three management factors (W, S, and N) interacted to shape soil moisture and salinity. Higher irrigation increased soil water storage, which is beneficial for crop water supply in the arid environment, but it also increased salt input and salt accumulation when brackish water was applied. Similar patterns have been observed in arid northwestern China, where larger irrigation amounts can increase crop productivity partly by moving salts deeper into the soil profile [30]. Lower irrigation reduced soil moisture replenishment, but it also reduced salt input, especially when moderate-salinity water was used. The MDI system may have reduced salinity stress by moving salts laterally away from drip emitters and limiting salt concentration around roots [31]. Thus, W1 did not necessarily impose severe stress when it was combined with S2 and adequate N. A previous study [32] similarly reported that irrigation water of 3–4 dS m^−1^ improved maize water use efficiency by reducing excessive transpiration without strongly limiting photosynthesis. In the present study, S2 (3.5 dS m^−1^) did not produce severe root-zone salt stress under W1, supporting the agronomic feasibility of controlled brackish–water irrigation.

N application also changed soil water and salt dynamics. Adequate N supply (N2) supported plant growth and root development under lower irrigation, allowing the crop to extract and use soil water more effectively. Min et al. [33] reported a similar effect in a cotton field, where moderate N fertilization improved cotton growth and water use efficiency at salinity up to 4.6 dS m^−1^. We observed a significant N effect on SWS in 2024, which did not appear in 2023. Such a year-specific effect of N on SWS may be associated with differences in accumulated precipitation and crop water uptake between the two seasons. Greater or differently distributed rainfall can change soil water redistribution under MDI and alter the extent to which N-induced changes in crop growth and root water uptake are reflected in measured SWS. Therefore, the significant N effect in 2024 should be interpreted as a seasonal response rather than a stable long-term pattern.

Our results indicated that N should not be over-applied in saline irrigation systems. According to Jiang et al. [32], N rates above 270 kg ha^−1^ under brackish irrigation increased alkaline ions and soil salinity, which inhibited N uptake and reduced yield. In our study, compared with the N1 level, SSA under the N3 level significantly increased by 19.36% and 25.00% in 2023 and 2024, respectively (Table 2). This response may be partly attributed to the additional ionic load introduced by excessive N input and to fertilizer-induced acidification during N transformation, which can release native cations and enhance the redistribution of soluble salts [34,35].

### 3.2. Interactive Effects of Irrigation, Salinity, and Nitrogen on Soil Enzymes

Soil moisture was identified as the most positive driver of enzyme activity in this study. The activities of URE, ALP, PRO, and SUC all increased with higher irrigation amounts, consistent with the stimulation of microbial processes by improved hydration. Because microbial biomass and community data were not measured, we interpret these enzyme responses as indicators of potential biochemical functioning rather than direct evidence of microbial community change [36,37]. Adequate water supply likely enhanced microbial growth and substrate diffusion, which in turn supported enzyme production and function. However, elevated soil salinity had the opposite effect and suppressed nearly all measured enzyme activities. This response is consistent with previous reports from saline soils, where high salt content inhibits enzymes involved in carbon (C), N, and P cycling by disrupting microbial physiology [15,17]. URE and PRO are directly related to N transformation, because URE catalyzes urea hydrolysis and PRO participates in the decomposition of proteinaceous organic N. Their inhibition under high salinity, therefore, suggests weaker N mineralization and lower N supply capacity for maize growth [23,24]. ALP reflects organic P mineralization potential and is often enhanced when P demand is high, whereas SUC is associated with labile C turnover and microbial energy supply [38,39]. The simultaneous decline in these enzymes under S3 indicates that high salinity weakened multiple nutrient cycling processes rather than affecting only one pathway.

Additionally, the four enzymes did not respond uniformly to N addition. PRO activity increased with higher N application rate, whereas ALP and SUC activities declined at the highest N rate, and URE activity peaked at N2 before decreasing at the N3 level. Notably, higher irrigation can also buffer the negative effect of excessive N on URE by improving soil hydration, diluting local fertilizer concentration, and enhancing substrate diffusion. This explains why the decline from N2 to N3 was less pronounced under W2 than under W1. These patterns likely reflect differences in substrate demand and enzyme regulation. N addition can stimulate microbial growth and demand for mineralizable N sources, which may increase proteolytic enzyme production when N is supplied at a moderate rate [40]. By contrast, high mineral N availability can reduce microbial investment in C acquisition enzymes such as SUC [41]. For phosphatase, abundant N without a matching increase in P may alter microbial nutrient allocation and reduce ALP production. In addition, N-induced acidification may also shift soil pH away from the optimum for ALP activity [42]. The nonlinear URE response suggests that urea substrate availability limited activity at low N, whereas ammonium accumulation and pH shifts may have reduced URE activity at very high N [43].

The significant N × S interaction for enzyme activity indicates that salinity changed the way soil enzymes responded to N supply (Figure 2). Under brackish water irrigation, an appropriate input of N appeared to counteract some salt stress, thereby maintaining enzyme activity. Yet excessive N did not restore enzyme activity under severe salinity and may have intensified ionic stress by increasing salt accumulation [44]. This result agrees with the context-dependent responses reported by Li et al. [45]. Within an appropriate range, N addition can relieve nutrient limitation in saline soils; beyond that range, it may aggravate osmotic stress or nutrient imbalance [46]. Overall, responses of these four enzyme activities help explain the observed maize yield changes in a biochemical way. Reduced URE, PRO, ALP, and SUC activities could limit nutrient supply during maize growth and thereby contribute to lower yield.

### 3.3. Grain Yield Formation Mechanism Analysis

The SEM results showed that SWS had a positive effect on yield (total effect: 0.228), whereas SSA had a negative effect mainly by reducing GN (path coefficient: −0.65, *p* < 0.05; Figure 3a). The ANOVA results further showed that irrigation amount did not significantly affect 1000-grain weight in either year, whereas grain number was strongly affected by W, S, N, and their interactions (Tables S1 and S2). This suggests that maize under moderate stress tended to maintain the filling of established kernels, while the number of kernels formed was more sensitive to water–salt–nitrogen conditions. This pattern is reasonable because grain number in maize is largely determined during tasseling, flowering, and early kernel set, which are highly sensitive to water deficit and salinity-induced osmotic stress [47,48]. In particular, salt stress during early kernel development can restrict assimilate supply and carbohydrate metabolism in young ovaries and developing kernels, thereby increasing kernel abortion and reducing final grain number [49,50]. Although sufficient irrigation generally promotes kernel set and grain filling, W2 (675 mm) did not maximize yield in this experiment. Instead, the best yield occurred under W1 (450 mm) combined with moderate salinity and N. This may be due to excessive irrigation reducing yield benefits by increasing nutrient leaching, limiting soil aeration, or promoting vegetative growth at the expense of reproductive allocation [2,18]. N availability affected yield mainly by supporting GW and photosynthetic capacity during the grain filling stage [51]. Within a suitable range, higher N can increase kernel size because the crop can accumulate more protein and starch. The SEM showed a positive influence of N on GW, but its total effect on grain yield was negative (Figure 3b). Thus, N was necessary for high yield but adding too much N under saline irrigation did not increase grain yield synchronously.

In the current study, grain yield peaked at the N2 (330 kg ha^−1^) level, whereas further N addition produced little benefit or a decline. Under sufficient irrigation (W2), N3 (435 kg ha^−1^) even reduced yield, likely because excessive vegetative growth diverted assimilates away from grain development. Sun et al. [52] also noted that luxury N uptake can delay maturity and increase transpiration without increasing grain output. However, under relatively lower irrigation, adequate N application helped maintain N uptake and leaf photosynthesis under stress, improving grain formation per unit water [53]. This interaction explains the strong performance of W1S2N2: W1 supplied enough water for critical growth stages, S2 imposed only mild osmotic stress, and N2 prevented nutrient limitation during biomass production and grain filling. The W × S × N interaction can therefore be interpreted as a balance among water supply, salt stress, nutrient availability, and biochemical functioning [19,30,36,54]. Higher irrigation improved soil hydration and enzyme activity, but when the applied water contained salts, it also increased salt input. Moderate salinity and moderate N maintained relatively high enzyme activity and yield, whereas high salinity combined with excessive N produced chemical stress that could not be fully offset by higher irrigation. These non-additive responses explain why the best integrated treatment was not the highest water or highest N treatment, but W1S2N2. Yan et al. [18] also reported a comparable optimum for spring maize under drip irrigation, with peak yield at 450 mm irrigation and 300 kg N ha^−1^, and no further yield gain from over-irrigation or over-fertilization.

### 3.4. Implications and Limitations

These findings have practical implications for water–fertilizer management and brackish water utilization in arid and semi-arid regions. Although brackish water can help relieve freshwater shortages, it must be used with a reasonable irrigation regime to avoid salt accumulation and fertility decline [21,55,56]. Under MDI, seasonal irrigation of about 450 mm, N application of about 330 kg ha^−1^, and brackish irrigation salinity near 3.5 dS m^−1^ appeared suitable for maize production in Xinjiang. This combination balanced yield, IWP, and PFPN while limiting excessive salt accumulation and maintaining enzyme activity.

Although the optimal regime (W1S2N2) did not cause clear soil deterioration during the two-year experiment, longer use of saline irrigation requires continued monitoring. Salt accumulation under brackish water irrigation can be cumulative and may differ from short-term responses, especially when annual precipitation and winter leaching vary among years. Periodic leaching during the off-season or in wet years may be needed to move accumulated salts below the root zone, especially if salt build-up begins to appear. In addition, this study did not measure microbial biomass, microbial community composition, or functional genes. Therefore, the enzyme responses should be interpreted as potential indicators of soil biochemical functioning rather than direct evidence of microbial mechanisms. Future studies combining enzyme assays with microbial community and functional analyses would help clarify the biological pathways underlying W × S × N interactions. The main contribution of this study is to show that high maize yields can be achieved with brackish water when irrigation amount, salinity, and N rate are optimized together, rather than managed as independent inputs.

## 4. Materials and Methods

### 4.1. Site Description

**Figure 5:**
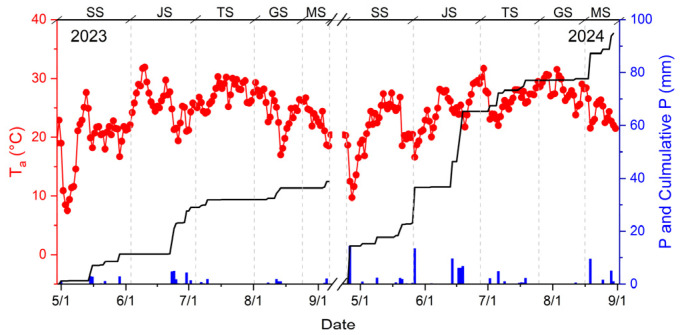
Daily average air temperature (Ta), precipitation (P), and cumulative P during the maize growing season (May to September) in 2023 and 2024.

The field experiment was conducted during two consecutive maize growing seasons, 2023 and 2024, at the Key Laboratory of Modern Water-saving Irrigation in Xinjiang Province, northwest China (85.59° E, 44.19° N, 412 m a.s.l.). The site has cold winters, hot, dry summers, and low rainfall, which are typical of an arid continental climate. Daily air temperature and precipitation during the two growing seasons are shown in Figure 5. The soil is classified as sandy loam according to the USDA soil taxonomy [57]. Mean soil bulk density was 1.45 g cm^−3^ at 0–40 cm and 1.61 g cm^−3^ at 40–100 cm.

**Figure 6:**
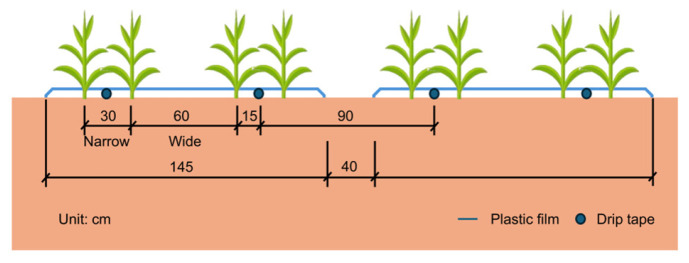
Schematic diagram of maize planting patterns and mulched drip irrigation tape layout.

During the two-year field investigation, the locally dominant maize cultivar ‘Fengyu 33’ was sown on 29 April 2023 and 23 April 2024, and harvested on 6 September 2023 and 30 August 2024, respectively. The irrigation system used mulched drip irrigation, with two drip tapes placed 90 cm apart under a 1.45 m-wide plastic film. Each tape irrigated two maize rows, and the emitter flow rate was 2.6 L h^−1^. An alternating wide-narrow row pattern was used according to the mulch seeder: wide rows were spaced 60 cm apart, narrow rows were spaced 30 cm apart, and plants were spaced 20 cm within rows (Figure 6). The theoretical planting density was 82,500 plants ha^−1^.

### 4.2. Experimental Design and Field Management

**Figure 7:**
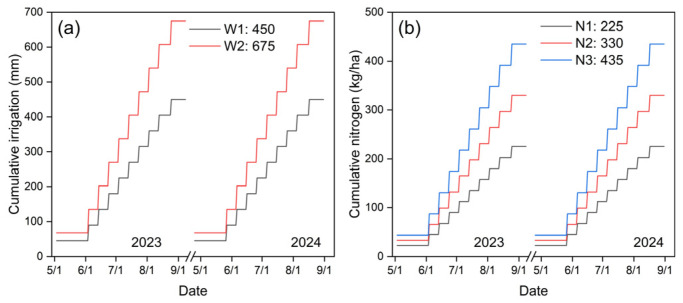
Cumulative irrigation amount (**a**) and nitrogen application (**b**) during the maize growing season for different treatments.

The experiment included three factors: irrigation amount (W), irrigation salinity (S), and nitrogen application rate (N). The irrigation amount had two levels: 450 mm (W1) and 675 mm (W2). Irrigation salinity had three levels: 1.3 dS m^−1^ (0.85 g NaCl L^−1^, S1), 3.5 dS m^−1^ (3 g NaCl L^−1^, S2), and 5.7 dS m^−1^ (5 g NaCl L^−1^, S3). S1 represents the local irrigation water, whereas S2 and S3 were prepared by adding industrial salt (NaCl > 99.10%) to the local water to reach the target salinity levels. The ionic composition of local water was 10.32 meq L^−1^ Na^+^, 0.64 meq L^−1^ K^+^, 1.38 meq L^−1^ Mg^2+^, 1.42 meq L^−1^ Ca^2+^, 7.31 meq L^−1^ Cl^−^, 1.24 meq L^−1^ SO_4_^2−^, and 1.08 meq L^−1^ HCO_3_^−^, with a pH of 7.31. N was applied as urea at rates of 22, 300, and 435 kg ha^−1^ for N1, N2, and N3, respectively. There was a total of 18 treatment combinations, each replicated three times. Potassium and phosphorus fertilizers were applied at 60 kg ha^−1^ and 100 kg ha^−1^, respectively, in all treatments. Total irrigation and fertilizer inputs were distributed across maize growth stages according to local practice [58]. The first irrigation was applied on the sowing day to support germination and early seedling establishment. After the jointing stage, irrigation and fertilization were scheduled systematically. Ten irrigation and fertilization events were applied during each growing season, and cumulative inputs for 2023 and 2024 are shown in Figure 7.

### 4.3. Field Sampling and Laboratory Analysis

#### 4.3.1. Soil Water Content (SWC), Soil Salt Content (SSC), and Soil Total Nitrogen (TN)

At the end of each maize growth stage, to cover the main effective root zone of maize and the principal soil layer affected by water–salt migration under MDI [59,60,61], soil samples were collected with a soil auger from 0 to 100 cm at 10 cm intervals. Samples from each depth were divided into two subsamples for SWC and SSC measurements. SWC (%) was measured by oven drying at 105 °C for more than 24 h until a constant weight. For SSC (g kg^−1^), soil electrical conductivity (EC_1:5_, dS m^−1^) was first measured in a 1:5 soil–water extract [62,63]. The EC_1:5_ values were then converted to SSC using the calibration equation established for this study site [57]:

(1) SSC = 1.0366 EC_1:5_

Soil water storage and soil salt accumulation were calculated using the following equations [64]:

(2) $$\mathrm{SWS}\;=\;\sum_{i=1}^{n}w_{i}\;\times {\;\gamma }_{i}\;\times \;h_{i}\;\times \;10$$

(3) $$SSA\;=\;\sum_{i=1}^{n}s_{i}\;\times {\;\gamma }_{i}\;\times \;h_{i}\;\times \;0.01$$

where SWS is the soil water storage (mm), SSA is the soil salt accumulation (kg ha^−1^), wi is the SWC (%), s_i_ is the SSC (g kg^−1^), γ_i_ is the soil bulk density at the ith soil layer (g cm^−3^), and h_i_ is the soil depth (cm).

After harvest, soil from 0–20 cm was collected for TN measurement using the Dumas combustion method with a carbon-nitrogen analyzer (CN-802, VELP, Monza, Italy) [50]. The remaining fresh soil was used to determine the activities of urease (EC 3.5.1.5, URE), alkaline phosphatase (EC 3.1.3.1, ALP), proteinase (EC 3.2.1.8, PRO), and sucrase (EC 3.2.1.48, SUC). Fresh soil samples were transported to the laboratory in a refrigerator immediately after collection. Visible roots and stones were removed, and the samples were gently passed through a 2 mm sieve. Subsamples for enzyme analysis were stored at 4 °C and analyzed as soon as possible to minimize changes in enzyme activity.

#### 4.3.2. Soil Enzyme Activities

URE activity was measured using the sodium phenate-sodium hypochlorite colorimetric method [65,66]. Fresh sieved soil (10 g) was mixed with 10 mL citrate buffer (pH 6.7) and 0.5 mL toluene to inhibit microbial activity, and then incubated at 37 °C for 24 h. After a 10% urea solution was added, the samples were incubated for another 2 h. The reaction was stopped before phenol, sodium, and sodium hypochlorite solutions were added for color development, and absorbance was measured at 578 nm. ALP activity was determined by p-nitrophenyl phosphate (PNPP) hydrolysis [67]. Soil aliquots (5 g) were treated with toluene and Tris-HCl buffer (pH 9.4), followed by the addition of the PNPP substrate solution. After 1 h of incubation at 37 °C with shaking, the reaction was stopped by centrifugation. The supernatant was alkalinized with 0.5 M NaOH (final pH > 10), and absorbance was measured at 405 nm. PRO activity was measured using the casein-Folin method [68]. Soil samples (2 g) were incubated with phosphate buffer (pH 7.4) and 2% casein solution at 37 °C for 2 h. After the reaction was stopped with trichloroacetic acid and the samples were centrifuged, the supernatant was mixed with sodium carbonate and Folin reagent. SUC activity was measured using the 3,5-dinitrosalicylic acid colorimetric assay (DNS), with modifications based on T/NAIA 010-2020 [69]. Soil (5.00 g) was incubated with 5 mL of 8% sucrose in 0.2 M phosphate buffer (pH 5.5) at 37 °C for 24 h, with buffer-only controls included. The terminated supernatants were mixed 1:1 (*v*/*v*) with DNS reagent, boiled for 5 min, and diluted to 10 mL. Absorbance at 540 nm was converted to reducing sugar concentration using a glucose standard curve.

#### 4.3.3. Grain Yield, Irrigation Water Productivity (IWP), and Partial Factor Productivity of Nitrogen (PFPN)

At maize maturity, three sampling points were randomly selected in each plot. Three plants were collected at each point to determine grain yield and yield components (1000-grain weight, GW; grain number, GN). The harvested plants were air-dried, threshed, and weighed. Irrigation water productivity (IWP, kg mm^−1^) and partial factor productivity of nitrogen (PFPN, kg kg^−1^) were calculated as grain yield divided by the total irrigation water input and the total N fertilizer input, respectively [63,70,71,72].

### 4.4. Statistical Analysis

Three-way analysis of variance (ANOVA) was used to test the main and interactive effects of irrigation amount (W), irrigation salinity (S), and nitrogen application rate (N), using the ‘tidyverse’ package in R software (version 4.3.2). Variable differences among treatments were evaluated using Tukey’s test at *p* < 0.05. To further explore the mechanisms driving maize grain yield and to quantify the total effects of each variable, a structural equation model (SEM) was established using the ‘plspm’ package. Goodness of fit (GOF) was used to assess overall model performance and predictive capability, with higher GOF indicating better fit.

A comprehensive evaluation framework was used to compare the overall performance of the water–salinity–nitrogen treatments. Principal component analysis (PCA), entropy weighting, and TOPSIS were combined to extract key soil indicators, assign objective weights, and rank treatments. To reduce systematic differences caused by interannual environmental variation, year-adjusted values were calculated for all indicators. These adjusted values were then averaged at the treatment level to obtain cross-year estimates. Following the principle of removing random year effects in mixed-effects models [73,74], the adjustment was calculated as:

(4) $$X_{\mathrm{ij}}^{\mathrm{adj}}\;=\;X_{\mathrm{ij}}\;-\;\left({\bar{X}}_{\cdot j}^{\mathrm{year}}-{\bar{X}}_{\cdot \cdot j}\right)$$

where $X_{\mathrm{ij}}^{\mathrm{adj}}$ is the year-adjusted (cross-year robust) value, $X_{\mathrm{ij}}$ is the original observed value of treatment i in year, and ${\bar{X}}_{\cdot j}^{\mathrm{year}}$ is the mean of indicator j across all treatments within that year, ${\bar{X}}_{\cdot \cdot j}$ is the overall multi-year mean of the indicator.

After year adjustment, all year-adjusted variables were normalized before PCA, entropy weighting, and TOPSIS. PCA was first conducted using the adjusted soil indicators, and the first three principal components with eigenvalues greater than 1 were retained. The selected soil indicators, together with maize yield, IWP, and PFPN, were then used to build the comprehensive evaluation matrix. Indicator weights were calculated using the entropy weight method [75]. TOPSIS was applied to calculate the closeness coefficient (C_i_) [76]:

(5) $$C_{i}\;=\;\frac{d_{i}^{-}}{d_{i}^{+}\;+\;d_{i}^{-}}$$

where $d_{i}^{+}$ is the Euclidean distance from treatment i to the positive ideal solution, and $d_{i}^{-}$ is the Euclidean distance to the negative ideal solution.

## 5. Conclusions

This study evaluated how irrigation amount, irrigation salinity, and N application rate jointly affected soil water–salt–nitrogen dynamics, soil enzyme activity, maize yield, and resource use efficiency under mulched drip irrigation in arid northwestern China. Higher irrigation increased soil water storage, but it also increased salt accumulation, especially when saline water was used. Moderate irrigation and N application balanced soil moisture and salinity, creating favorable conditions that supported enzyme activity and maize growth. Maize grain yield, irrigation water productivity (IWP), and partial factor productivity of nitrogen (PFPN) were all influenced by both the main effects and interactions of irrigation, salinity, and N. The structural equation model (SEM) revealed that soil salt accumulation was the main constraint on yield formation and acted primarily by reducing grain number rather than grain weight. The integrated evaluation identified an irrigation amount of 450 mm, an irrigation salinity of 3.5 dS m−1, and an N application rate of 330 kg ha^−1^ as the best combination for achieving high yield and IWP while maintaining high PFPN under two-year experimental conditions. Moreover, this recommendation should be limited to similar climate and soil conditions to those in Xinjiang. The longer-term application of brackish water at 3.5 dS m^−1^ will require continued salinity monitoring and, where necessary, leaching management to avoid soil degradation.

## Figures and Tables

**Table 1:** Summary of ANOVA significance levels for the effects of irrigation amount (W), irrigation salinity (S), and nitrogen application rate (N) on soil water storage (SWS) at the depths of 0–40 cm, 40–100 cm, and 0–100 cm during the maize growing season in 2023 and 2024, respectively.

Factors	2023			2024		
	0–40 cm	40–100 cm	0–100 cm	0–40 cm	40–100 cm	0–100 cm
Irrigation level (W)						
W1	90.32 ± 7.64 b	126.72 ± 17.44 b	217.04 ± 23.89 b	95.87 ± 8.06 b	134.24 ± 18.63 b	230.11 ± 25.21 b
W2	108.95 ± 9.9 a	167.34 ± 13.29 a	276.29 ± 20.56 a	114.84 ± 10.24 a	177.55 ± 14.92 a	292.39 ± 22.4 a
Nitrogen application rate (N)						
N1	99.46 ± 13.61 a	146.64 ± 26.00 a	246.10 ± 37.99 a	107.22 ± 13.33 a	158.94 ± 28.18 a	266.16 ± 39.97 a
N2	100.79 ± 12.33 a	148.84 ± 25.38 a	249.63 ± 36.54 a	102.01 ± 12.58 b	150.70 ± 26.03 b	252.71 ± 37.03 b
N3	98.65 ± 12.61 a	145.6 ± 25.49 a	244.25 ± 36.94 a	106.84 ± 13.27 a	158.05 ± 27.69 a	264.89 ± 39.67 a
Irrigation salinity (S)						
S1	93.09 ± 11.09 c	138.08 ± 27.59 c	231.17 ± 37.51 c	98.64 ± 11.64 c	147.03 ± 29.54 c	245.67 ± 39.73 c
S2	99.71 ± 11.23 b	146.5 ± 21.65 b	246.21 ± 31.38 b	105.74 ± 11.61 b	154.85 ± 23.74 b	260.59 ± 33.59 b
S3	106.11 ± 12.79 a	156.51 ± 23.94 a	262.62 ± 35.59 a	111.68 ± 13.13 a	165.81 ± 25.76 a	277.50 ± 37.87 a
ANOVA (F-value)						
W	458.26 ***	585.86 ***	695.55 ***	461.55 ***	573.45 ***	695.18 ***
N	2.06 ^ns^	1.29 ^ns^	1.97 ^ns^	14.39 ***	8.34 ***	13.17 ***
S	74.65 ***	40.3 ***	65.37 ***	72.87 ***	36.32 ***	60.6 ***
W × N	0.09 ^ns^	0.07 ^ns^	0.07 ^ns^	0.14 ^ns^	0.41 ^ns^	0.33 ^ns^
W × S	1.68 ^ns^	1.56 ^ns^	1.85 ^ns^	1.78 ^ns^	1.46 ^ns^	1.78 ^ns^
N × S	0.27 ^ns^	0.05 ^ns^	0.06 ^ns^	0.20 ^ns^	0.11 ^ns^	0.13 ^ns^
W × N × S	0.03 ^ns^	0.04 ^ns^	0.01 ^ns^	0.30 ^ns^	0.08 ^ns^	0.10 ^ns^

Note: W1 and W2 indicate irrigation amounts of 450 mm and 675 mm, respectively; S1, S2, and S3 indicate irrigation salinity levels of 1.3 dS m^−1^, 3.5 dS m^−1^, and 5.7 dS m^−1^, respectively; N1, N2, and N3 indicate nitrogen application rates of 225 kg ha^−1^, 330 kg ha^−1^, and 435 kg ha^−1^, respectively. Values are presented as mean ± standard deviation, where values not sharing a common letter are significantly different at *p*  <  0.05 as determined by Tukey’s test. *** indicate significant levels at *p* < 0.001; “ns” indicates no significant difference. The same below.

**Table 2:** Summary of ANOVA significance levels for the effects of irrigation amount (W), irrigation salinity (S), and nitrogen application rate (N) on soil salt accumulation (SSA) at soil depths of 0–40 cm, 40–100 cm, and 0–100 cm during the maize growing season in 2023 and 2024, respectively.

Factors	2023			2024		
	0–40 cm	40–100 cm	0–100 cm	0–40 cm	40–100 cm	0–100 cm
Irrigation level (W)						
W1	25.94 ± 10.91 a	36.78 ± 11.35 b	62.72 ± 20.75 b	28.21 ± 11.60 a	40.62 ± 12.63 b	68.83 ± 22.53 b
W2	27.11 ± 13.39 a	41.36 ± 16.57 a	68.47 ± 29.35 a	29.65 ± 13.81 a	45.30 ± 17.86 a	74.95 ± 30.89 a
Nitrogen application rate (N)						
N1	25.09 ± 11.53 b	34.85 ± 13.20 c	59.94 ± 23.63 b	26.47 ± 12.20 b	36.75 ± 14.04 c	63.22 ± 25.02 c
N2	26.50 ± 12.61 ab	38.80 ± 13.93 b	65.30 ± 25.56 b	29.54 ± 12.24 a	43.87 ± 13.94 b	73.41 ± 24.96 b
N3	27.99 ± 12.41 a	43.56 ± 14.71 a	71.54 ± 26.27 a	30.77 ± 13.52 a	48.26 ± 16.66 a	79.03 ± 29.17 a
Irrigation salinity (S)						
S1	14.08 ± 3.81 c	28.63 ± 3.85 c	42.72 ± 6.84 c	15.85 ± 4.19 c	31.92 ± 4.62 c	47.77 ± 7.92 c
S2	26.6 ± 5.64 b	37.81 ± 8.16 b	64.41 ± 12.64 b	29.38 ± 6.24 b	41.25 ± 8.88 b	70.63 ± 13.86 b
S3	38.90 ± 9.62 a	50.76 ± 17.09 a	89.66 ± 25.43 a	41.55 ± 10.01 a	55.72 ± 18.60 a	97.27 ± 27.09 a
ANOVA (F-value)						
W	2.05 ^ns^	13.92 ***	8.86 **	2.86 ^ns^	12.97 ***	9.28 **
N	4.22 *	16.83 ***	12.08 ***	9.01 ***	26.59 ***	21.18 ***
S	309.82 ***	109.36 ***	197.61 ***	303.68 ***	113.37 ***	202.50 ***
W × N	0.40 ^ns^	2.43 ^ns^	0.56 ^ns^	0.15 ^ns^	2.91 ^ns^	0.89 ^ns^
W × S	4.43 *	6.70 **	6.42 **	3.59 *	6.28 **	5.78 **
N × S	0.33 ^ns^	1.40 ^ns^	0.68 ^ns^	0.32 ^ns^	2.17 ^ns^	1.30 ^ns^
W × N × S	0.70 ^ns^	0.78 ^ns^	0.12 ^ns^	0.75 ^ns^	0.72 ^ns^	0.07 ^ns^

Values are presented as mean ± standard deviation, where values not sharing a common letter are significantly different at *p*  <  0.05 as determined by Tukey’s test. *, **, *** indicate significant levels at *p* < 0.05, *p* < 0.01, and *p* < 0.001, respectively; “ns” indicates no significant difference.

**Table 3:** Effects of irrigation amount (W), irrigation salinity (S), and nitrogen application rate (N) on the 1000-grain weight, grain number, grain yield, irrigation water productivity (IWP), and partial factor productivity of nitrogen (PFPN) in 2023.

Treatment	2023					2024				
	1000-Grain Weight (g)	Grain Number	Grain Yield (kg ha^−1^)	IWP (kg mm^−1^)	PFPN (kg kg^−1^)	1000-Grain Weight (g)	Grain Number	Grain Yield (kg ha^−1^)	IWP (kg mm^−1^)	PFPN (kg kg^−1^)
W1 S1 N1	293.74 ± 8.48 abc	466.7 ± 9.31 d	15,134.54 ± 266.56 f	33.63 ± 0.59 d	67.26 ± 1.18 a	296.46 ± 5.9 ab	472.6 ± 5.46 g	15,173.83 ± 134.45 i	33.72 ± 0.3 f	67.44 ± 0.6 c
W1 S1 N2	297.55 ± 5.95 abc	481.4 ± 7.96 cd	15,671.52 ± 167.16 cde	34.83 ± 0.37 bc	47.49 ± 0.51 d	300.65 ± 6.85 ab	488.9 ± 8.79 ef	15,918.65 ± 76.14 g	35.37 ± 0.17 d	48.24 ± 0.23 g
W1 S1 N3	308.73 ± 9.2 ab	468 ± 8.88 d	15,467.2 ± 166.25 def	34.37 ± 0.37 cd	35.56 ± 0.38 f	314.55 ± 38.79 ab	474.4 ± 7.63 fg	16,160.69 ± 28.33 f	35.91 ± 0.06 c	37.15 ± 0.07 j
W1 S2 N1	299.44 ± 6.56 abc	485.3 ± 9.84 cd	15,438.53 ± 44.1 ef	34.31 ± 0.1 cd	68.62 ± 0.2 a	295.4 ± 13.44 ab	488.4 ± 1.74 efg	15,624.68 ± 80.43 h	34.72 ± 0.18 e	69.44 ± 0.36 a
W1 S2 N2	305.17 ± 3.4 abc	528.3 ± 4.13 b	17,663.86 ± 38.33 a	39.25 ± 0.09 a	53.53 ± 0.12 b	322.41 ± 32.79 ab	525.7 ± 6.97 ab	18,355.76 ± 90.15 a	40.79 ± 0.2 a	55.62 ± 0.27 d
W1 S2 N3	300.59 ± 6.89 abc	494 ± 7.17 c	15,918.85 ± 95.72 bc	35.38 ± 0.21 b	36.6 ± 0.22 f	310.45 ± 12.38 ab	499.5 ± 8.07 de	16,794.24 ± 88.75 cd	37.32 ± 0.2 b	38.61 ± 0.2 i
W1 S3 N1	298.25 ± 6.19 abc	325.2 ± 11.44 hi	10,504.23 ± 116.29 ij	23.34 ± 0.26 ghi	46.69 ± 0.52 d	293.26 ± 5.18 ab	300.1 ± 1.01 j	9531.34 ± 49.87 m	21.18 ± 0.11 k	42.36 ± 0.22 h
W1 S3 N2	297.3 ± 9.96 abc	337.5 ± 6.32 gh	10,866.53 ± 98.9 i	24.15 ± 0.22 f	32.93 ± 0.3 g	288.86 ± 23.36 ab	309.4 ± 9.56 ij	9679.25 ± 82.32 m	21.51 ± 0.18 k	29.33 ± 0.25 l
W1 S3 N3	310.17 ± 5.74 ab	305.8 ± 3.97 i	10,272.18 ± 100.78 j	22.83 ± 0.22 hij	23.61 ± 0.23 i	333 ± 40.53 a	279.4 ± 5.53 k	10,076.28 ± 27.8 l	22.39 ± 0.06 j	23.16 ± 0.06 n
W2 S1 N1	286.04 ± 10 c	493.8 ± 5.62 c	15,294.59 ± 169 ef	22.66 ± 0.25 ij	67.98 ± 0.75 a	284.32 ± 12.87 b	498.7 ± 3.06 de	15,355.91 ± 73.91 i	22.75 ± 0.11 j	68.25 ± 0.33 b
W2 S1 N2	292.66 ± 8.19 bc	499.8 ± 4.05 c	16,130.95 ± 102.25 b	23.9 ± 0.15 fg	48.88 ± 0.31 c	295.1 ± 4.38 ab	518.6 ± 6.63 bc	16,574.23 ± 128.65 de	24.55 ± 0.19 hi	50.22 ± 0.39 f
W2 S1 N3	294.61 ± 6.39 abc	545.7 ± 8.96 ab	17,475.05 ± 236.93 a	25.89 ± 0.35 e	40.17 ± 0.54 e	299.72 ± 7.34 ab	517.6 ± 3.76 bc	16,800.95 ± 70.81 c	24.89 ± 0.1 h	38.62 ± 0.16 i
W2 S2 N1	285.99 ± 5.76 c	492 ± 4.17 c	15,241.25 ± 61.36 f	22.58 ± 0.09 j	67.74 ± 0.27 a	282.07 ± 11.31 b	497.5 ± 5.12 de	15,197.47 ± 100.74 i	22.51 ± 0.15 j	67.54 ± 0.45 bc
W2 S2 N2	298.01 ± 8.84 abc	554.2 ± 13.02 a	17,565.32 ± 166.87 a	26.02 ± 0.25 e	53.23 ± 0.51 b	306.25 ± 11.04 ab	537.4 ± 9.11 a	17,824.13 ± 46.31 b	26.41 ± 0.07 g	54.01 ± 0.14 e
W2 S2 N3	295.69 ± 10.79 abc	497.7 ± 5.04 c	15,880.01 ± 103.79 bcd	23.53 ± 0.15 fgh	36.51 ± 0.24 f	300.55 ± 5.99 ab	507.6 ± 3.6 cd	16,522.14 ± 17.57 e	24.48 ± 0.03 i	37.98 ± 0.04 i
W2 S3 N1	313.33 ± 9.43 a	346.5 ± 3.16 fg	11,758.03 ± 195.58 h	17.42 ± 0.29 l	52.26 ± 0.87 b	311.81 ± 11.54 ab	319.5 ± 7.32 i	10,789.38 ± 79.88 k	15.98 ± 0.12 m	47.95 ± 0.36 g
W2 S3 N2	305.7 ± 6.59 abc	362.8 ± 11.2 f	12,113.92 ± 157.27 h	17.95 ± 0.23 l	36.71 ± 0.48 f	297.77 ± 9.25 ab	345.1 ± 7.36 h	11,129.05 ± 74 j	16.49 ± 0.11 l	33.72 ± 0.22 k
W2 S3 N3	308.31 ± 9.33 ab	387.2 ± 10.16 e	12,819.04 ± 362.22 g	18.99 ± 0.54 k	29.47 ± 0.83 h	309.46 ± 13.02 ab	322.2 ± 5.16 i	10,798.54 ± 177.33 k	16.00 ± 0.26 m	24.82 ± 0.41 m
ANOVA (F-value)										
W	2.51 ^ns^	213.79 ***	320.50 ***	13,122.28 ***	239.98 ***	2.26 ^ns^	209.82 ***	289.49 ***	51,004.57 ***	256.12 ***
S	8.07 **	2295.83 ***	4727.39 ***	5478.74 ***	4597.59 ***	0.70 ^ns^	5349.56 ***	28,276.82 ***	30,408.48 ***	24,753.20 ***
N	3.43 *	46.66 ***	204.47 ***	211.98 ***	12,089.49 ***	4.02 *	78.27 ***	1032.23 ***	1041.98 ***	36,834.22 ***
W × S	6.11 **	20.67 ***	129.68 ***	648.97 ***	114.42 ***	0.80 ^ns^	19.91 ***	351.92 ***	2671.50 ***	345.33 ***
W × N	0.70 ^ns^	26.51 ***	57.97 ***	58.09 ***	15.56 ***	0.74 ^ns^	4.85 *	5.93 **	22.59 ***	8.47 ***
S × N	1.66 ^ns^	25.48 ***	95.97 ***	96.21 ***	162.85 ***	1.89 ^ns^	9.83 ***	345.58 ***	365.71 ***	610.05 ***
W × S × N	1.03 ^ns^	13.51 ***	12.50 ***	8.53 ***	6.85 ***	0.78 ^ns^	1.81 ^ns^	19.35 ***	41.68 ***	36.10 ***

Values are presented as mean ± standard deviation, where values not sharing a common letter are significantly different at *p*  <  0.05 as determined by Tukey’s test. *, **, *** indicate significant levels at *p* < 0.05, *p* < 0.01, and *p* < 0.001, respectively; “ns” indicates no significant difference.

## Data Availability

The datasets generated and/or analyzed during the current study are not publicly available as they form part of ongoing research. Access to the data may be granted upon reasonable request, subject to the completion of the associated studies.

## Supplementary Materials

The following supporting information can be downloaded at: https://www.mdpi.com/article/10.3390/plants15142165/s1, Table S1: Effects of irrigation amount (W), irrigation salinity (S), and nitrogen application rate (N) on the 1000-grain weight, grain number, grain yield, irrigation water productivity (IWP), and partial factor productivity of nitrogen (PFPN) in 2023; Table S2: Effects of irrigation amount (W), irrigation salinity (S), and nitrogen application rate (N) on the 1000-grain weight, grain number, grain yield, irrigation water productivity (IWP), and partial factor productivity of nitrogen (PFPN) in 2024; Table S3: Variable loadings of three principal components; Table S4: Entropy weights of SSA_0–100_, ALP, PRO, Yield, IWP, and PFPN.

## Abbreviations

The following abbreviations are used in this manuscript:

ALP	Alkaline phosphatase
ANOVA	Analysis of variance
C	Carbon
DNS	3, 5-dinitrosalicylic acid
EA	Enzyme activity
EC	Electrical conductivity
GN	Grain number
GOF	Goodness of fit
GW	1000-grain weight
IWP	Irrigation water productivity
K	Potassium
MDI	Mulched drip irrigation
N	Nitrogen
ns	No significant difference
NUE	Nitrogen use efficiency
P	Phosphorus
PCA	Principal component analysis
PFPN	Partial factor productivity of nitrogen
PNPP	p-nitrophenyl phosphate
PRO	Protease
S	Irrigation salinity
SEM	Structural equation model
SSS_0–40_	Soil salt storage at 0–40 cm
SSS_40–100_	Soil salt storage at 40–100 cm
SSS_0–100_	Soil salt storage at 0–100 cm
SUC	Sucrase
SWS_0–40_	Soil water storage at 0–40 cm
SWS_40–100_	Soil water storage at 40–100 cm
SWS_0–100_	Soil water storage at 0–100 cm
TN	Total nitrogen
TOPSIS	Technique for order preference by similarity to an ideal solution
URE	Urease
USDA	United states department of agriculture
W	Irrigation amount
