# Supplementary material for: Interactive Effects of Irrigation Amount, Irrigation Salinity, and Nitrogen Rate on Soil Enzyme Activities and Maize (Zea mays L.) Yield Under Mulched Drip Irrigation
Source: Plants (Basel). 2026 Jul 14;15(14):2165. doi: 10.3390/plants15142165 (PMC13417438; doi:10.3390/plants15142165)
Supplement: Supplementary file 1 [file plants-15-02165-s001.zip › plants-4398057-supplementary.pdf]

Table S1. Effects of irrigation amount (W), irrigation salinity (S), and nitrogen application rate (N) on the 1000-grain weight, grain number, grain yield, irrigation water productivity (IWP), and partial factor productivity of nitrogen (PFPN) in 2023.

**Factors**	**1000-grain weight (g)**	**Grain number**	**Grain yield (kg ha^{-1})**	**IWP (kg mm^{-1})**	**PFPN (kg kg^{-1})**
Irrigation level (W)					
W1	301.21±8.03a	432.47±81.49b	14104.16±2660.87b	31.34±5.91a	45.81±14.78b
W2	297.82±11.62a	464.41±75.37a	14919.8±2112.19a	22.1±3.13b	48.1±13.16a
Nitrogen application rate (N)					
N1	296.13±11.67b	434.92±73.22c	13895.2±2051.92c	25.66±6.38c	61.76±9.12a
N2	299.4±7.87ab	460.67±84.35a	15002.02±2686.24a	27.68±7.39a	45.46±8.14b
N3	303.02±9.53a	449.73±82.61b	14638.72±2465.72b	26.83±6.23b	33.65±5.67c
Irrigation salinity (S)					
S1	295.56±9.83b	492.57±28.22b	15862.31±825.91b	29.21±5.32b	51.22±12.79b
S2	297.48±8.69b	508.58±26.13a	16284.64±1001.27a	30.18±6.59a	52.7±13.3a
S3	305.51±9.12a	344.17±27.75c	11388.99±954.39c	20.78±2.82c	36.94±10.12c
ANOVA (F-value)					
W	2.51^{ns}	213.79^{***}	320.50^{***}	13122.28^{***}	239.98^{***}
N	8.07^{**}	2295.83^{***}	4727.39^{***}	5478.74^{***}	4597.59^{***}
S	3.43^{*}	46.66^{***}	204.47^{***}	211.98^{***}	12089.49^{***}
W * S	6.11^{**}	20.67^{***}	129.68^{***}	648.97^{***}	114.42^{***}
W * N	0.70^{ns}	26.51^{***}	57.97^{***}	58.09^{***}	15.56^{***}
S * N	1.66^{ns}	25.48^{***}	95.97^{***}	96.21^{***}	162.85^{***}
W * S * N	1.03^{ns}	13.51^{***}	12.50^{***}	8.53^{***}	6.85^{***}

Table S2. Effects of irrigation amount (W), irrigation salinity (S), and nitrogen application rate (N) on the 1000-grain weight, grain number, grain yield, irrigation water productivity (IWP), and partial factor productivity of nitrogen (PFPN) in 2024.

Factors	1000-grain weight (g)	Grain number	Grain yield (kg ha^{-1})	IWP (kg mm^{-1})	PFPN (kg kg^{-1})
Irrigation level (W)					
W1	306.12±24.62a	426.49±95.42b	14146.08±3276.26b	31.44±7.28a	45.71±15.39b
W2	298.56±12.96a	451.58±89.50a	14554.64±2734.73a	21.56±4.05b	47.02±14.23a
Nitrogen application rate (N)					
N1	293.89±13.34b	429.47±87.78b	13612.1±2544.78c	25.15±7.00c	60.5±11.31a
N2	301.84±18.49ab	454.18±94.42a	14913.51±3408.60a	27.52±8.46a	45.19±10.33b
N3	311.29±23.42a	433.45±98.40b	14525.47±2991.22b	26.83±7.73b	33.39±6.88c
Irrigation salinity (S)					
S1	298.47±17.3a	495.13±19.7b	15997.38±613.89b	29.53±5.71b	51.65±12.73b
S2	302.85±19.13a	509.35±18.28a	16719.74±1148.96a	31.04±7.10a	53.87±12.73a
S3	305.7±23.14a	312.62±21.57c	10333.97±627.35c	18.93±2.88c	33.56±9.27c
ANOVA (F-value)					
W	2.26^{ns}	209.82^{***}	289.49^{***}	51004.57^{**}*	256.12^{***}
N	0.70^{ns}	5349.56^{***}	28276.82^{***}	30408.48^{***}	24753.20^{***}
S	4.02^{*}	78.27^{***}	1032.23^{***}	1041.98^{***}	36834.22^{***}
W * S	0.80^{ns}	19.91^{***}	351.92^{***}	2671.50^{***}	345.33^{***}
W * N	0.74^{ns}	4.85^{*}	5.93^{**}	22.59^{***}	8.47^{***}
S * N	1.89^{ns}	9.83^{***}	345.58^{***}	365.71^{***}	610.05^{***}
W * S * N	0.78^{ns}	1.81^{ns}	19.35^{***}	41.68^{***}	36.10^{***}

Table S3. Variable loadings of three principal components.

Variables	PC1(5.17)	PC2(4.52)	PC3(1.26)
TN	0.370	0.128	-0.382
$SSA_{0\text{-}40}$	0.430	0.108	0.100
$SSA_{40\text{-}100}$	0.439	0.057	-0.035
$SSA_{0\text{-}100}$	**0.442**	0.080	0.022
$SWS_{0\text{-}40}$	0.267	-0.384	0.029
$SWS_{40\text{-}100}$	0.284	-0.372	0.004
$SWS_{0\text{-}100}$	0.278	-0.377	0.013
URE	-0.154	-0.321	-0.446
ALP	-0.154	**-0.422**	0.211
PRO	-0.111	-0.339	**-0.573**
SUC	-0.017	-0.372	0.519

TN: total nitrogen; SSA0-40, SSA40-100, and SSA0-100: soil salt accumulation at the depth of 0-40 cm, 40-100 cm, and 0-100 cm; SWS0-40, SWS40-100, and SWS0-100: soil water storage at the depth of 0-40 cm, 40-100 cm, and 0-100 cm; URE: urease activity; ALP: alkaline phosphatase activity; PRO: protein activity; SUC: sucrase activity.

Table S4. Entropy weights of SSA0-100, ALP, PRO, Yield, IWP, and PFPN.

Indicator	Entropy weight
$SSA_{0\text{-}100}$	0.235
ALP	0.125
PRO	0.111
Yield	0.178
IWP	0.202
PFPN	0.149

SSA0-100: soil salt accumulation at the depth of 0-100 cm; ALP: alkaline phosphatase activity; PRO: protein activity; Yield: maize grain yield; IWP: irrigation water productivity; PFPN: partial factor productivity of nitrogen.
